# RIPCAL: a tool for alignment-based analysis of repeat-induced point mutations in fungal genomic sequences

**DOI:** 10.1186/1471-2105-9-478

**Published:** 2008-11-12

**Authors:** James K Hane, Richard P Oliver

**Affiliations:** 1 Australian Centre for Necrotrophic Fungal Pathogens, Faculty of Health Sciences, Murdoch University, South Street, Murdoch, 6150, Australia

## Abstract

**Background:**

Repeat-induced point mutation (RIP) is a fungal-specific genome defence mechanism that alters the sequences of repetitive DNA, thereby inactivating coding genes. Repeated DNA sequences align between mating and meiosis and both sequences undergo C:G to T:A transitions. In most fungi these transitions preferentially affect CpA di-nucleotides thus altering the frequency of certain di-nucleotides in the affected sequences. The majority of previously published *in silico *analyses were limited to the comparison of ratios of pre- and post-RIP di-nucleotides in putatively RIP-affected sequences – so-called RIP indices. The analysis of RIP is significantly more informative when comparing sequence alignments of repeated sequences. There is, however, a dearth of bioinformatics tools available to the fungal research community for alignment-based RIP analysis of repeat families.

**Results:**

We present RIPCAL , a software tool for the automated analysis of RIP in fungal genomic DNA repeats, which performs both RIP index and alignment-based analyses. We demonstrate the ability of RIPCAL to detect RIP within known RIP-affected sequences of *Neurospora crassa *and other fungi. We also predict and delineate the presence of RIP in the genome of *Stagonospora nodorum *– a Dothideomycete pathogen of wheat. We show that RIP has affected different members of the *S. nodorum *rDNA tandem repeat to different extents depending on their genomic contexts.

**Conclusion:**

The RIPCAL alignment-based method has considerable advantages over RIP indices for the analysis of whole genomes. We demonstrate its application to the recently published genome assembly of *S. nodorum*.

## Background

Over 100 fungal genome sequences have been obtained or are in the pipeline [1] and next-generation sequencing technologies will further accelerate the accumulation of data over the next decade. This rapidly growing array of sequence information presents many new challenges for analysis. There is an urgent need to develop and implement efficient tools to describe features of new genomes. Repeat-induced point mutation (RIP) is one such area of fungal biology requiring efficient analytical tools. RIP is an irreversible genome defence mechanism first detected in *Neurospora crassa *[2,3] and subsequently in *Magnaporthe grisea *[4,5], *Podospora anserina *[6] and *Leptosphaeria maculans *[7]. RIP is believed to be a defence against transposons, rendering them inactive and protecting sexual progeny from the expression of transposon genes.

Direct experimental observation of RIP requires both that the fungal species can be crossed under laboratory conditions and that the strain can be transformed with multiple copies of a transgene. Very few fungal species are amenable to such analysis and these procedures are slow in all cases. RIP-like processes can also be detected by *in-silico *analysis of repeated elements in whole or partial genomic sequences. Prior examples include *Aspergillus fumigatus *[8], *Fusarium oxysporum *[9-11], *Aspergillus nidulans *[12], *Microbotryum violaceum *[13], *Magnaporthe oryzae *[14], *Aspergillus niger *[15] and *Penicillium chysogenum *[15]. We now have the opportunity to detect and measure RIP *in silico *from genomic sequences of diverse species.

RIP involves transitions from C:G to T:A nucleotides in pairs of duplicated sequences during the dikaryotic phase between mating and meiosis [2,3]. RIP changes are scattered throughout both sequences where pairs share more than ~80% identity [16] and are over 400 bp in length [17]. C:G transitions are not random within affected sequences. Particular CpN dinucleotides are preferentially altered over others (Table 1). In *N. crassa*, CpA di-nucleotides were preferentially altered [18]. Thus a strong bias towards CpA to TpA changes (or TpG to TpA in the complementary strand) was observed. This resulted in a relative decrease in CpA and TpG and a corresponding increase in TpA di-nucleotides within RIP-affected sequences. These changes in di-nucleotide frequencies can be used to identify RIP-affected repeats by measuring the ratios of pre-RIP and post-RIP di-nucleotides within a set of repeated sequences. This generates a single statistic called a "RIP index" (plural: RIP indices). High frequencies of post-RIP and low frequencies of pre-RIP di-nucleotides are straightforward to detect by this method and useful for identifying RIP-affected sequences. The RIP indices TpA/Apt and (CpA+TpG)/(ApC+GpT), originally developed by Margolin *et al *[19], are commonly used to detect RIP *in silico *[8,12,19,20]. TpA/ApT is the simplest index and measures the frequency of TpA RIP products with correction for false positives due to A:T rich regions. Higher values of TpA/ApT indicate a stronger RIP response. The index (CpA+TpG)/(ApC+GpT) is similar in principle to TpA/ApT but measures the depletion of the RIP targets CpA and TpG. In this case lower values of (CpA+TpG)/(ApC+GpT) are indicative of stronger RIP.

**Table 1:** The four possible CpN→TpN di-nucleotide RIP mutations and their reverse complements which form the basis for comparisons to determine the dominant form of RIP mutation in both alignment-based and statistical analyses.

RIP Mutation	Reverse Complement*
CpA→TpA	TpG→TpA
CpC→TpC	GpG→GpA
CpG→TpG	CpG→CpA
CpT→TpT	ApG→ApA

(*The forward orientation is often referred to in the text, however should be understood to include the reverse complement unless specified otherwise.)

RIP-indices are simple to calculate and do not require complete knowledge of the genome sequence or repeat families. They are also applicable to heavily mutated repeat families for which an alignment is not possible or questionable. However, RIP indices are insensitive tools which obscure many interesting features of RIP. These include the direction of RIP changes (i.e. which sequence is closer to the ancestral precursor of the RIP-affected sequence), the degree of RIP along the length of repeat alignments and differences in RIP profiles between members of the repeat class.

As RIP operates on aligned sequences, these questions are better answered using an alignment-based approach. Alignment-based analysis of RIP involves the multiple alignment of a repeat family and counting RIP mutations along the alignment for all sequences. This method has been previously used to identify RIP within the Ty1 transposon family of *Microbotryum violaceum *using the software tool Sequencher. Such manual calculation of RIP as was used by Hood *et al *[13] does not lend itself to whole genome RIP analysis. To enable a thorough, facile and automated analysis of RIP in the plethora of new fungal genomes, we have developed the free software tool RIPCAL (available at . RIPCAL incorporates both RIP index and alignment-based methods. Its capabilities are demonstrated with examples taken from *de novo*-defined repeat families of the recently published *Stagonospora nodorum *genome, a major fungal pathogen of wheat [21,22].

## Results

### Validation of RIP detection by the alignment-based method

The RIPCAL alignment-based method was applied to both the 5S rDNA repeat family of *Neurospora crassa*, which is reportedly free from RIP mutation due to its short sequence length [17], and to the Tad1 transposons of *N. crassa*, which are reported to be heavily prone to CpA→TpA RIP mutation [23]. The 5S rDNA and Tad1 repeat families served as negative and positive controls for RIP respectively. Analysis showed low levels of RIP mutation among 5S rDNAs, whereas high levels of RIP mutation were detected amongst Tad1 transposons as expected (Additional file 1). Interestingly, while CpA↔TpA changes were highly increased in the Tad1 family, these were overshadowed by a major increase in CpT↔TpT mutation, which has not been previously detected [23]. This may be due to the fact that the former study compared Tad1 sequences between different strains of *Neurospora crassa*, whereas this comparison was restricted to all repeats within a single strain.

### Identification of the dominant CpN to TpN di-nucleotide mutation in RIP-affected sequences

*De novo *RIP analysis of a fungal repeat unit first requires the identification of the most affected CpN di-nucleotides. The MATE transposon repeat family of *Aspergillus nidulans *and the Ty1 Copia-like transposon family of the Basidiomycete *Microbotryum violaceum *were analysed by RIPCAL. *A. nidulans *MATE repeats are reported to exhibit a dual preference for CpG→TpG and CpA→TpA RIP mutation in descending order of magnitude [24]. The Ty1 repeats of *M. violaceum *were reported to exhibit a strong preference for CpG→TpG di-nucleotide RIP mutation [13]. High levels of CpG→TpG and CpA→TpA RIP mutation were detected in the MATE transposons (Additional file 2). RIPCAL also detected the CpG→TpG bias in the Ty1 repeats of *M. violaceum *(Additional file 1). Hood *et al *have reported preferential mutation of the tri-nucleotide TpCpG to TpTpG in Ty1 [13], however RIPCAL is not currently designed to detect a tri-nucleotide RIP bias.

### Di-nucleotide frequency and index analysis of RIP mutation in *Stagonospora nodorum*

**Figure 1:**
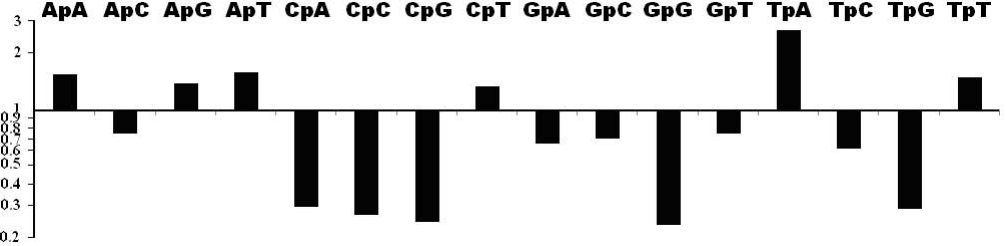
**Fold changes in di-nucleotide abundances for all repeat families of *Stagonospora nodorum *compared to non-repetitive control sequence on a Log_10 _scale**. This conforms to the expected pattern associated with classical CpA→TpA type RIP mutation: high TpA and low CpA and TpG abundances.

RIPCAL di-nucleotide frequency analyses of the previously identified de novo repeat families Molly, Pixie, Elsa, Y1 (rDNA repeat), R8, R9, R10, R22, R25, R31, R37, R38, R39, R51, X0, X3, X11, X12, X15, X23, X26, X28, X35, X36, X48 and X96 [21] of the *S. nodorum *genome were performed and indicated depletion of the CpA, CpC, CpG, GpG and TpG di-nucleotide targets of RIP-mutation (Figure 1, Additional file 2). Of the RIP di-nucleotide products, only TpA showed a corresponding increase. This suggests that CpA to TpA is the dominant form of CpN→TpN di-nucleotide mutation in repeats of *S. nodorum*, as observed in *N. crassa *and *P. anserina *[6,20]. This is corroborated by RIP index analysis. RIP indices for TpA/ApT were well in excess of *S. nodorum *non-repetitive control sequences indicating high frequencies of the TpA RIP product in the repeat families. The (CpA+TpG)/(ApC+GpT) index was below control levels indicating depletion of the CpA and TpG RIP targets in the repeats. Both dinucleotide frequency and RIP index analyses strongly indicated that the mutation of CpA to TpA was the dominant form of di-nucleotide RIP mutation in the repeat families of *S. nodorum *(Table 2, Additional file 2).

**Table 2:** Analysis of *Stagonospora nodorum* repeat families for evidence of RIP ranked by CpA↔TpA dominance.

Repeat Family	$\frac{TpA}{ApT}$	$\frac{CpA+TpG}{ApC+GpT}$	CpA↔TpA Dominance	Alignment Length	Description/Homology
R8	1.70 ± 0.03	0.74 ± 0.02	2.96	9548	Ubiquitin conjugating enzyme
X0	1.75 ± 0.02	0.49 ± 0.02	2.13	4103	Non LTR transposon
R10	1.76 ± 0.06	0.56 ± 0.05	1.91	1360	Unknown
R9	1.99 ± 0.03	0.48 ± 0.02	1.88	4483	Non LTR transposon
X48	1.35 ± 0.11	1.26 ± 0.13	1.82	275	Sub-telomeric repeat
rDNA Non-tandem	2.68 ± 0.18	0.69 ± 0.04	1.50	9938	Non-array rDNA repeats ≥ 1 kb
X35	1.76 ± 0.07	0.58 ± 0.07	1.50	1185	Sub-telomeric repeat
MOLLY	1.90 ± 0.06	0.40 ± 0.04	1.21	1946	Mariner-like transposon
R22	1.73 ± 0.08	0.27 ± 0.04	1.20	710	Sub-telomeric repeat
X26	1.70 ± 0.03	0.52 ± 0.02	1.16	5034	Sub-telomeric repeat/Transposon remnant
R31	1.65 ± 0.04	0.44 ± 0.04	0.99	3119	Unknown
X36	1.97 ± 0.18	0.44 ± 0.10	0.89	516	Unknown
X96	1.87 ± 0.19	0.56 ± 0.18	0.87	319	Unknown
ELSA	1.67 ± 0.04	0.46 ± 0.05	0.86	5273	Copia-like transposon
X11	2.04 ± 0.03	0.38 ± 0.02	0.83	7570	Gypsy-like transposon
X28	2.22 ± 0.13	0.39 ± 0.03	0.83	1975	Unknown
PIXIE	1.84 ± 0.07	0.36 ± 0.03	0.77	1918	Mariner-like transposon
X12	2.06 ± 0.07	0.33 ± 0.04	0.67	2059	Gypsy-like transposon
X3	1.91 ± 0.03	0.74 ± 0.01	0.63	10673	Helicase
X15	1.87 ± 0.04	0.33 ± 0.02	0.61	6437	Sub-telomeric repeat/Gypsy-like transposon
R39	1.92 ± 0.08	0.30 ± 0.03	0.59	2102	Unknown
rDNA Tandem	2.08 ± 0.09	0.94 ± 0.02	0.53	9938	rDNA repeats in tandem array
R37	1.85 ± 0.03	0.28 ± 0.02	0.49	2264	Mariner-like transposon
R51	1.93 ± 0.07	0.27 ± 0.03	0.47	870	Unknown
X23	1.85 ± 0.09	0.31 ± 0.03	0.45	613	Unknown
rDNA Short	3.55 ± 0.39	0.25 ± 0.03	0.26	280*	Non-array rDNA repeats < 1 kb
R25	2.16 ± 0.15	0.31 ± 0.03	0.25	3407	Transposon remnant
R38	2.10 ± 0.18	0.24 ± 0.05	0.20	391	Unknown
Non-Repetitive Control	0.83 ± 0.01	1.25 ± 0.00	N/A	N/A	Genomic regions not corresponding to repeat matches

Two RIP index scores are given within a 95% confidence interval. The alignment-based comparison of CpA↔TpA RIP-mutation is used to give dominance score. CpA↔TpA dominance is a numerical indicator of frequency of that mutation over other CpN↔TpN mutations as described in the methods. Values for the rDNA repeat are sub-classified according to physical location and length. The length of the alignment is also given. (*rDNA short alignments are a subset of the full-length rDNA alignment.)

### Alignment-based analysis of RIP mutation in *Stagonospora nodorum*

Repeat families of *S. nodorum *were aligned and scanned for RIP-like di-nucleotide changes using RIPCAL. RIP mutation statistics for all repeat families of *S. nodorum *are summarised in Additional file 2. Alignment-based analysis indicated that the dominant form of CpN-targeted RIP mutation in *S. nodorum *repeats was CpA to TpA as observed by index analysis. High levels of CpT to TpT mutation were also observed in some repeat classes (Additional file 2).

In this analysis we introduce a statistic called 'RIP dominance'. RIP dominance is the ratio of a particular CpN↔TpN RIP mutation over the sum of the other 3 alternative CpN↔TpN mutations within a multiple alignment (or sub-alignment). This was used to determine the relative strength of CpA to TpA type RIP mutations in *S. nodorum *(Table 2).

**Figure 2:**
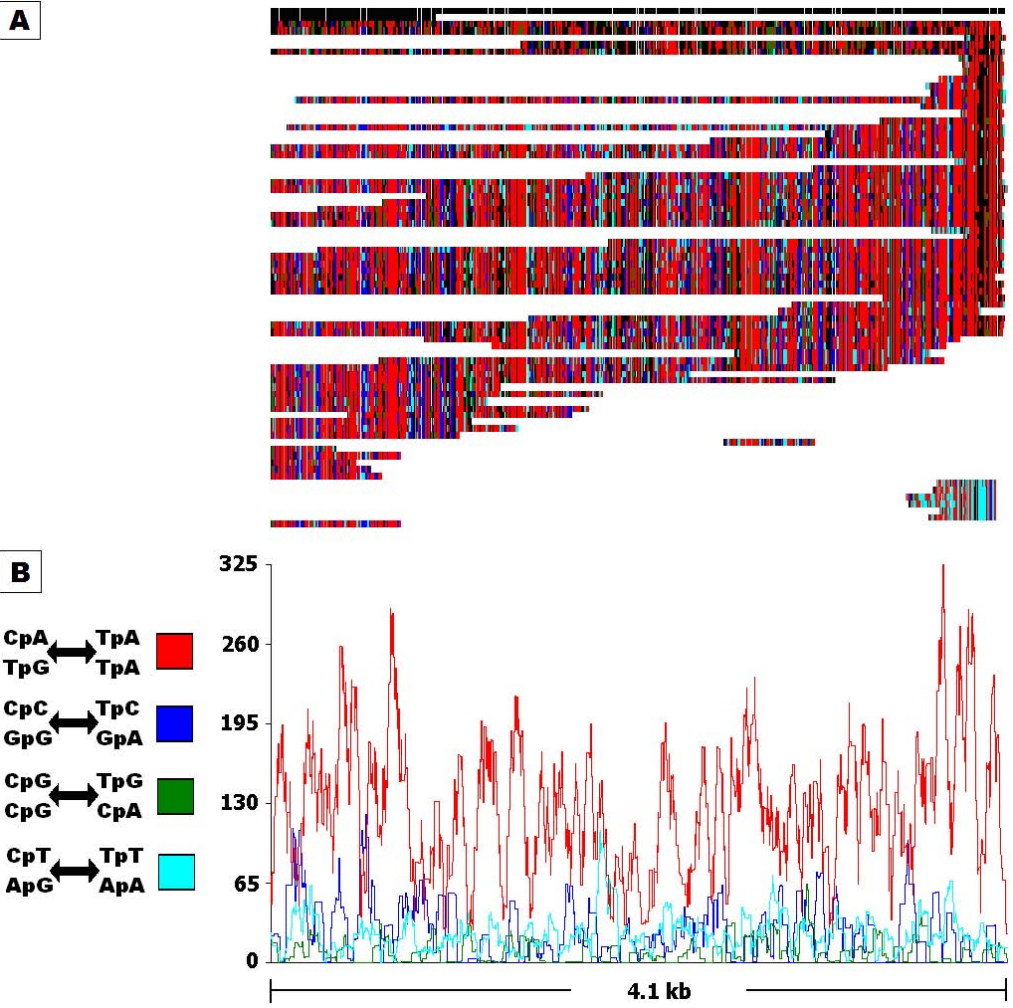
**RIPCAL analysis of the X0 repeat family of *Stagonospora nodorum*, representative of a repeat family exhibiting strongly dominant classical CpA→TpA type RIP mutation**. A) multiple alignment of the putative transposon repeat family X0 compared to highest G:C content model. Incomplete repeated regions are typical for repeat family alignments illustrated by the blocks in white in panel A. Black = match; grey = mismatch; white = gap. Mismatches corresponding to selected di-nucleotide changes are coloured as indicated. B) Overall RIP mutation frequency graph over a 50 bp scanning window, corresponding to the alignment above, demonstrating the overall dominance of the CpA↔TpA mutation over other CpN↔TpN mutations for the X0 repeat family.

RIPCAL analysis of the XO repeat family of predicted non-LTR transposons is shown in Figure 2. The alignment (Figure 2A) displays the range of repeat sizes, sequence coverages and locations of RIP mutation for individual repeats. The repeat with the highest total G:C content was chosen as the least RIP-mutated model for comparison to all aligned sequences. CpN↔TpN di-nucleotide changes are colour-coded and show that CpA to TpA changes far outweighed all other CpN to TpN di-nucleotide mutations. Figure 2B shows the same data summarised as a rolling frequency graph. The RIP dominance for CpA↔TpA mutation in XO was 2.13, meaning that the CpA↔TpA mutation was more than twice as frequent as the sum of CpC, CpG and CpT-targeted RIP mutations. Each repeat element in this family showed a relatively equal degree of RIP. A slight tendency towards higher RIP incidence was found towards the ends of the alignment. XO appears to be a simple repeat unit which is highly and evenly RIP-affected.

**Figure 3:**
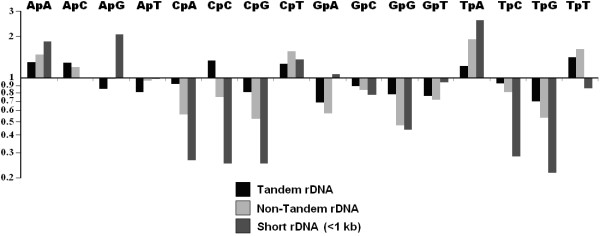
**Fold changes in di-nucleotide abundances between *Stagonospora nodorum *rDNA repeat sub-categories**. Tandem (black), non-tandem (light-grey) and short < 1 kb (dark grey) on a Log_10 _scale. Tandem rDNA repeats exhibit lesser variations in TpA, CpA and TpG counts, therefore are less RIP-affected than non-tandem and short < 1 kb rDNA repeats.

The *S. nodorum *rDNA repeat family provided a more complex example. *S. nodorum *Y1/rDNA repeats are located within a large tandem array on scaffold 5 and as non-tandem remnants scattered elsewhere throughout the genome. The non-tandem remnants were sub-divided into those longer or shorter than 1 kb. rDNA sub-classes differed markedly from the non-repetitive control by changes in di-nucleotide frequency (Figure 3). Tandem rDNA repeats appeared to be the least RIP affected in terms of Cp(A/C/G) depletion and increases in TpA, followed by the non-tandem and short repeats. RIP index analysis showed a similar trend (Table 2). Tandem, non-tandem and short rDNA repeats had TpA/ApT index scores of 2.08, 2.68 and 3.55 respectively. These values were among the highest TpA/ApT scores of all repeat classes suggesting extreme RIP mutation. The (CpA + TpG)/(ApC + GpT) index gave a similar result. Tandem, non-tandem and short rDNA repeats scored 0.94, 0.69 and 0.25 respectively. These values were among the lowest for all repeat classes, again suggesting extreme RIP mutation in the rDNA repeat sub-classes. In all cases, the short rDNA repeats had particularly extreme scores, suggesting that these were the most RIP-affected.

**Figure 4:**
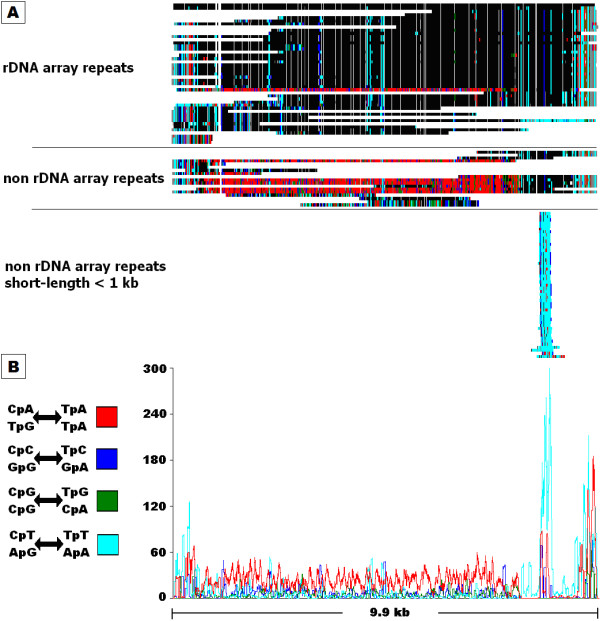
**RIPCAL analysis of the rDNA tandem repeat of *Stagonospora nodorum***. A) multiple alignment of the rDNA repeat family compared to highest G:C content model. Annotation is as for figure 3. Classical CpA↔TpA type RIP mutations are generally limited to full length rDNA-like repeats not located within the rDNA tandem array. One copy within the rDNA array exhibits RIP-like alterations. B) Overall RIP mutation frequency graph over a 50 bp scanning window, corresponding to the alignment above, demonstrating even dominance of CpA↔TpA changes in the non-array full-length repeats except near each end of the alignment.

When analysed by alignment (Figure 4), a more comprehensive picture emerged. The frequencies of CpN to TpN mutations (Figure 4B) indicated that CpA to TpA mutation was the dominant form of RIP mutation for the rDNA repeat family. However the distribution of RIP mutation within the alignment (Figure 4A) shows distinct differences in RIP profiles between the three rDNA sub-classes. The tandem rDNA repeats were generally unaffected by CpN-targeted mutation. Interestingly, a single tandem repeat was identified that had undergone extensive CpA to TpA changes. This proved to be the 5' terminal repeat within the rDNA array. The long non-tandem repeats were heavily affected by CpA to TpA RIP mutation, especially in the central regions. The short repeats showed no evidence of CpA to TpA RIP but did exhibit a high level of CpT to TpT RIP mutation. The CpA↔TpA RIP dominance score for non-tandem rDNA repeats was 1.5, whereas the tandem and short sub-classes had low scores of 0.53 and 0.26 (Table 2). This indicated heavy RIP mutation in non-tandem repeats and absence or low levels of RIP in tandem and short rDNA repeats.

## Discussion

The alignment-based method employed by RIPCAL is an efficient, accurate and reliable method of RIP detection and characterisation. RIPCAL successfully detected the presence and absence of RIP in the positive and negative *N. crassa *control sequences. RIPCAL also accurately determined the preferential CpN mutation bias in RIP-affected sequences. The CpG bias in Ty1 repeats of *M. violaceum *and the dual CpG and CpA bias in MATE repeats of *A. nidulans *were also identified consistent with previously published results [13,24].

Di-nucleotide frequency, RIP index and alignment-based analyses all indicated that CpA to TpA mutation was the dominant CpN-targeted mutation in the repeat families of *S. nodorum*. This preference is common to most known RIP-affected fungi. The high incidence of CpT to TpT mutation detected by alignment is less common, but has been observed in *Magnaporthe grisea *accompanying CpA-targeted mutation in RIP-affected sequences [4,5]. However high levels of CpT to TpT mutation within *S. nodorum *short rDNA repeats, which are presumably unaffected by RIP, suggest that CpT-targeted mutation may not related to RIP in *S. nodorum*. Further experimental evidence is required to confirm to relevance of CpT to TpT mutation to RIP in *S. nodorum *and other Fungi.

RIPCAL alignment-based analysis displays the physical distribution of RIP along an alignment as shown in for the X0 repeat family in Figure 2 and the Y1/rDNA repeat family in Figure 4. This allows detection of individual repeats with anomalous changes, such as the single RIP-affected tandem rDNA repeat (Figure 4A). The lack of CpA to TpA mutation within the tandem rDNA repeats adds further supporting evidence for RIP-resistance within the rDNA nucleolus organiser region (NOR) [2,25]. However, the RIP-affected tandem repeat, located at the terminus of the rDNA array suggests that protection from RIP within the NOR has a finite range.

**Figure 5:**
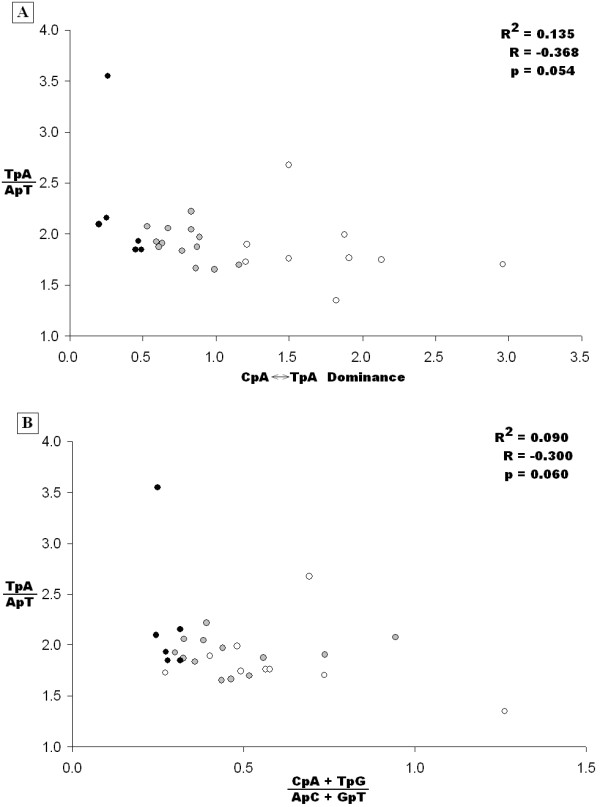
**Comparison of RIP indices with alignment-based RIPCAL comparisons for repeat families of *Stagonospora nodorum***. A) Comparison of TpA/ApT RIP index with the alignment-based CpA↔TpA dominance. A positive correlation was expected however was not observed. B) Comparison of the TpA/ApT and (CpA+TpG)/(ApC+GpT) RIP indices. A negative correlation would be expected. Repeat families exhibiting low levels of RIP by alignment based analysis are represented by black dots (CpA↔TpA dominance < 0.5); medium families are grey (0.5 ≤ CpA↔TpA dominance ≥ 1.2); and high are white (CpA↔TpA dominance > 1.2).

The close examination of the *S. nodorum *rDNA repeat sub-classes by alignment highlighted the poor performance of the RIP index based analyses. Differences in the extent of RIP mutation between DNA sub-classes by both TpA/ApT and (CpA + TpG)/(ApC + GpT) RIP indices were not as expected. This was particularly true for the short rDNA repeats which were predicted to exhibit the highest levels of RIP. Furthermore, both RIP indices predicted extreme RIP mutation in all sub-classes, which was only expected for the non-tandem rDNA repeats. Repeat order ranked by CpA↔TpA dominance is clearly different from that produced by either RIP index method (Table 2). The relationship between RIP index and CpA↔TpA dominance is shown in Figure 5A. There is no correlation (R^2 ^= 0.135) between the TpA/ApT RIP index and the CpA↔TpA dominance of *S. nodorum *repeats. Furthermore there was no significant correlation (R^2 ^= 0.090) between the two RIP indices (Figure 5B). We conclude that simple RIP indices are not reliable indicators of RIP mutation.

The length of a *S. nodorum *repeat class and the degree of RIP mutation did not appear to be related (Table 2). This was highlighted by X48, a short sub-telomeric repeat, which had a high CpA↔TpA dominance score of 1.82. Its length of 275 bp was well below the 400 bp length considered the minimum for RIP in *N. crassa *[17] and the 280 bp length of the S. nodorum short rDNA repeats (which do not display CpA to TpA changes). Alignment-based analysis predicted that sub-telomeric repeats were among the most RIP-susceptible. This may explain the high CpA↔TpA dominance of X48 as chromosome ends may be physically more accessible to the molecular RIP machinery. Alternatively, the X48 repeat may be recognised in conjunction with adjacent repeats as a single unit. Unlike the NOR, fungal telomeres do not appear to be immune to RIP. RIP-like changes have also been reported in the sub-telomeric gene *TLH *of *Magnaporthe oryzae *[14].

## Conclusion

We present RIPCAL as a versatile and efficient tool for the analysis of RIP which simplifies existing index-based analyses and adds alignment-based RIP analysis as a feasible alternative for whole genome analysis. These analyses highlight significant deficiencies in index-based methods of RIP detection. The alignment-based approach is biologically relevant and reveals novel features and predictions that can be tested experimentally in appropriate organisms. Sifting through the expected flood of fungal genome sequences for RIP-like phenomena may provide insights on fungal lifestyle, genomics and evolution.

## Methods

RIPCAL has multiple modes of operation involving different combinations of RIP index and alignment-based methods. RIPCAL can be run in either command-line or graphical modes and is Perl-based. It is also compiled as a Windows executable. Dependent on the analysis method, RIPCAL accepts sequence input in Fasta format, pre-aligned sequence input in Fasta or ClustalW format and repeat coordinate input in either version 2 or 3 GFF format. If pre-aligned input is not provided, RIPCAL can interface with a local installation of ClustalW [26]. Refer to Additional file 3 for more detailed information.

### RIP index analysis

Index analysis can proceed from either direct Fasta input, or from both Fasta and GFF coordinate inputs. RIP index analyses count frequencies of single nucleotides and the 16 possible di-nucleotide combinations, which are used to calculate RIP indices. Sequences were divided into sub-sequences of ≤ 100 bp length and di-nucleotide counts were normalised for N content by:

(1) $$\frac{Count\times (Length-Ncount)}{Length}$$

Where *Count *= di-nucleotide count, *Length *= length of sub-sequence and *Ncount *= count of unknown 'N' bases in sequence. Di-nucleotide counts were ignored where (*Length *- *Ncount*) < 10. The following indices have been published previously [19,27]:

(2) $$\frac{TpA}{ApT}$$

(3) $$\frac{CpA+TpG}{ApC+GpT}$$

Additional RIP indices that can be defined are of the form (CpN+NpG)/(TpN+NpA), which represents a ratio of conversion of pre-RIP di-nucleotides to post-RIP di-nucleotides, for the characteristic di-nucleotide mutation CpN→TpN and its reverse complement NpG→NpA (Table 1):

(4) $$\frac{CpA+TpG}{TpA}$$

(5) $$\frac{CpC+GpG}{TpC+GpA}$$

(6) $$\frac{CpG}{TpG+CpA}$$

(7) $$\frac{CpT+ApG}{TpT+ApA}$$

When using GFF input, RIP index data for repeat features was compared to a non-repetitive control family. If repeat family information is contained within the GFF input (via the target attribute) then this process was also separated by family. Fold changes between repeat families and the control were determined by ΔNpN = (repeat NpN count)/(control NpN count), where NpN represents any di-nucleotide combination.

### RIP index sequence scan

RIP indices are calculated over a user-defined window (default 200 bp). Using index thresholds as criteria for RIP, RIP-affected sub-regions were predicted and the output is given in GFF format. The default criteria for RIP within a sequence window were based on previously published data [19,27].

$$\frac{TpA}{ApT}\geq 0.89$$

$$\frac{CpT+ApT}{ApC+GpT}\leq 1.03$$

Where two windows meeting the above criteria overlap, the predicted sub-region was extended (Additional file 3). Sub-regions were subject to a minimum size threshold (default 300 bp) reflecting the existence of an experimentally observed size threshold for RIP [17]. Non-published indices were excluded by default, but can be employed as additional/replacement criteria using thresholds based on results obtained in this paper (Additional file 2). This method can be used to predict *de novo *ancient/non-repeated RIP-affected sequences. However, caution should be used with this method as the above threshold values are calibrated for RIP in *N. crassa*.

### Alignment-based analysis

RIPCAL's alignment-based analysis indicates the presence, type and location of a putatively RIP-generated mutation within each copy of a repeat family. The input is accepted as Fasta or as both Fasta and GFF inputs. "Repeat_region" features in the GFF input were aligned by family via ClustalW (Additional file 4, Additional file 5). The prevalence of internal direct repeats within repeat families can result in poor alignment. Therefore the ClustalW default parameters have been adjusted for fast alignment, pairwise window length = 50 and k-tuple word-size = 2 to improve repeat family alignment. In some cases custom alignment parameters or manual alignment curation was used and is recommended. Sequence-only inputs are also accepted as pre-aligned Fasta files. It is assumed for sequence-only inputs that all sequences belong to the same family.

Aligned sequences are compared to a model sequence which can be either a sequence with highest total G:C content in the alignment, the alignment consensus or a user-defined sequence. The default model selection method is highest total G:C content. As RIP mutations deplete the G:C content, this default is assumed to select the least RIP-affected sequence as the model. RIPCAL also provides alternative methods of model selection, one of which is to define a majority consensus of the aligned sequences. The degenerate nucleotide code is used if two or more nucleotides are present in equal frequency (Additional file 3). The third option is for the model to be user-defined. This would be appropriate if the non-RIP-affected sequence was known, as in the case of experimentally transformed strains.

Following alignment and choice of model, the mutation frequencies are compared along the alignment for each sequence. Where the consensus sequence is degenerate, the probability of mutation at that location is added to the total count. The final output is a repeat family alignment and corresponding RIP frequency graph in GIF format. A summary of RIP mutation type versus total sequence divergence per sequence is also generated based on the alignment.

### Validation of alignment-based RIP analysis

The alignment-based method was tested using the Tad1 transposon and 5S rDNA repeats from *Neurospora crassa *as positive and negative controls for detection of RIP mutation. These sequences [GenBank:L25662, GenBank:AF181821] were mapped to the *N. crassa *genome (release 7) [20] via RepeatMasker [28]. The genomic matches were compared via RIPCAL for RIP mutation. *Aspergillus nidulans *MATE transposon sequences [24] [GenBank:.BK001592, GenBank:.BK001593, GenBank:.BK0015924, GenBank:.BK001595, GenBank:.BK001596, GenBank:X78051] were compared via RIPCAL using MATE-9 [GenBank:.BK001592] as the model for comparison to test for detection of non-classical (non Cpa→TpA) RIP mutation. RIP mutation of Ty1 Copia-like transposons of *Mycrobotryum violaceum *[PopSet:55418573] was also analysed using the degenerate consensus model to observe RIP detection in sequences with a known tri-nucleotide mutation bias [13].

### RIP Analysis of *S. nodorum de novo *repeat families

Results herein use data from a recent survey of the genome of *S. nodorum *[21] (Additional file 4, Additional file 5). Repeat family genomic coordinates can be found in the supplementary data (Additional file 4). Repetitive sequences were identified *de novo *via RepeatScout [29], and filtered for ≥ 200 bp length; ≥ 10 × genomic match coverage and ≥ 75% identity. *De novo *repeats were mapped to the *S. nodorum *genome via RepeatMasker [28]. A total of 26 repeat families were identified, corresponding to roughly 4.5% of the assembled genomic sequence. The repeat families were aligned via ClustalW (Additional file 5). Some repeat families were predicted to be telomeric, where ≥ 85% of genomic matches resided on scaffold termini relative to overall localisation. The tandem rDNA repeats were defined by location within the rDNA tandem array on scaffold 5 [GenBank:CH445329] from base pair position 1310974 to 1594765. rDNA repeats at other locations were divided into non-tandem (≥ 1 kb) and short-length (< 1 kb) sub-families. The predicted repeat type was assigned based on BLAST versus NCBI and REPBASE [30]. RIP mutation 'dominance' represents the preponderance of a particular type of RIP di-nucleotide mutation relative to all other alternative forms of RIP mutation. CpA↔TpA dominance as referred to in Table 2 was determined by:

(8) $$\bar{\left(\frac{\left(CpA↔TpA\right)}{\left(CpC↔TpC)+(CpG↔TpG)+(CpT↔TpT\right)}\right)}$$

Other CpN↔TpN dominance equations (Additional file 2) were of a similar format to the one above (8).

### Time of Operation

All data was generated on a 2.99 GHz Dual-core ×64 Intel PC with 2 GB RAM. The combined run-time of the di-nucleotide and alignment-based analyses for the *S. nodorum *whole genome assembly was approximately 4 hours. Pre-aligned inputs with few sequences (i.e. < 20) can be expected to complete under a minute.

## Authors' contributions

JKH developed the RIPCAL software. JKH and RPO wrote the manuscript.

## Supplementary Material

Additional file 1 **Control Data**. Compressed (.zip) file containing data relevant to control tests with *Neurospora crassa *Tad1 and 5S rDNA repeats, containing RIPCAL graphical (.png), tabular text (.txt) and fasta (.fas) alignments of repeat family matches. Also contains files (.png, .txt and .fas) for the MATE repeats from *Aspergillus nidulans *and Ty1 transposons from *Microbotryum violaceum*. Click here for file

Additional file 2 **Supplementary Data Tables**. Excel (.xls) file with tabular data relating to RIPCAL analyses of *Stagonospora nodorum *repeats. Click here for file

Additional file 3 **Supplementary Methods**. Word (.doc) file explaining some of the methods in more detail. Click here for file

Additional file 4 **Snodorum Repeats**. GFF3 (.gff) file containing NCBI genomic accessions and coordinates of *Stagonospora nodorum *repeats used in this publication. Click here for file

Additional file 5 **Snodorum Alignments**. Compressed (.zip) file containing alignments for all *Stagonospora nodorum *repeat families in Fasta (.fas) format. Click here for file
