# Supplementary figures and images for: RIPCAL: a tool for alignment-based analysis of repeat-induced point mutations in fungal genomic sequences
Source: BMC Bioinformatics. 2008 Nov 12;9:478. doi: 10.1186/1471-2105-9-478 (PMC2621366; doi:10.1186/1471-2105-9-478)

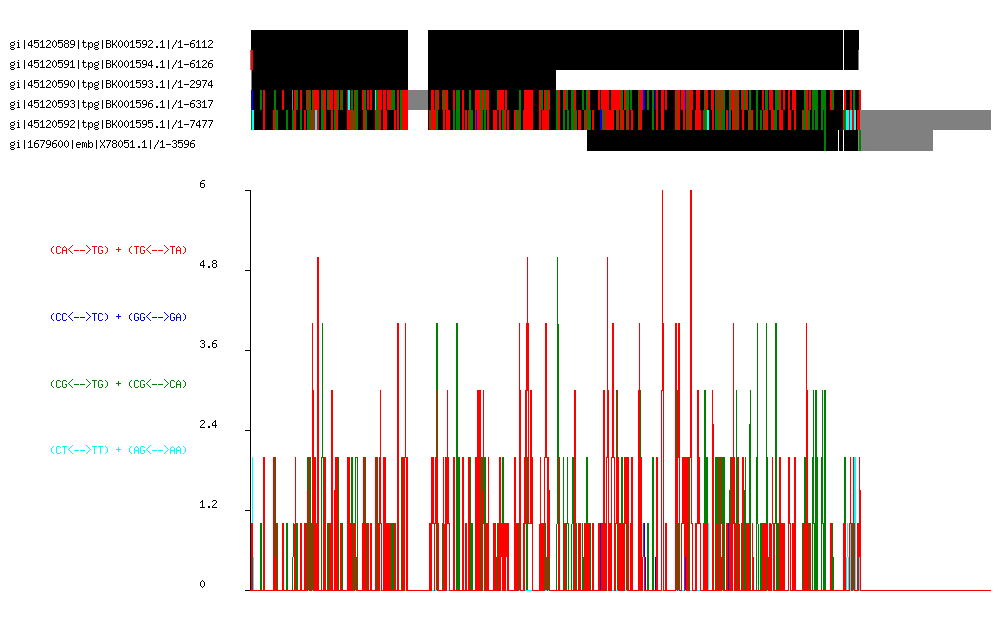

Supplement: Additional file 1 — Control Data. Compressed (.zip) file containing data relevant to control tests with Neurospora crassa Tad1 and 5S rDNA repeats, containing RIPCAL graphical (.png), tabular text (.txt) and fasta (.fas) alignments of repeat family matches. Also contains files (.png, .txt and .fas) for the MATE repeats from Aspergillus nidulans and Ty1 transposons from Microbotryum violaceum. [file 1471-2105-9-478-S1.zip › Supplementary Data_MATE_Emerica_nidulans.gif]

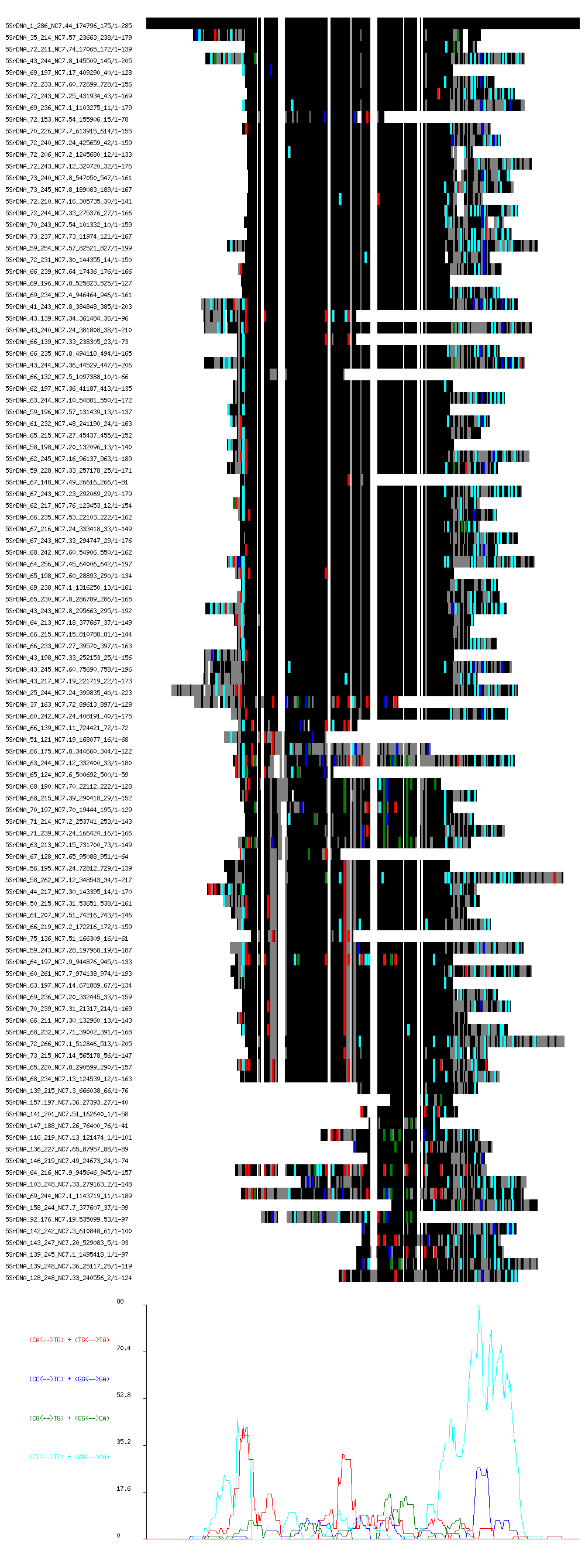

Supplement: Additional file 1 — Control Data. Compressed (.zip) file containing data relevant to control tests with Neurospora crassa Tad1 and 5S rDNA repeats, containing RIPCAL graphical (.png), tabular text (.txt) and fasta (.fas) alignments of repeat family matches. Also contains files (.png, .txt and .fas) for the MATE repeats from Aspergillus nidulans and Ty1 transposons from Microbotryum violaceum. [file 1471-2105-9-478-S1.zip › Supplementary Figure_5S rDNA alpha_Neurospora_crassa.gif]

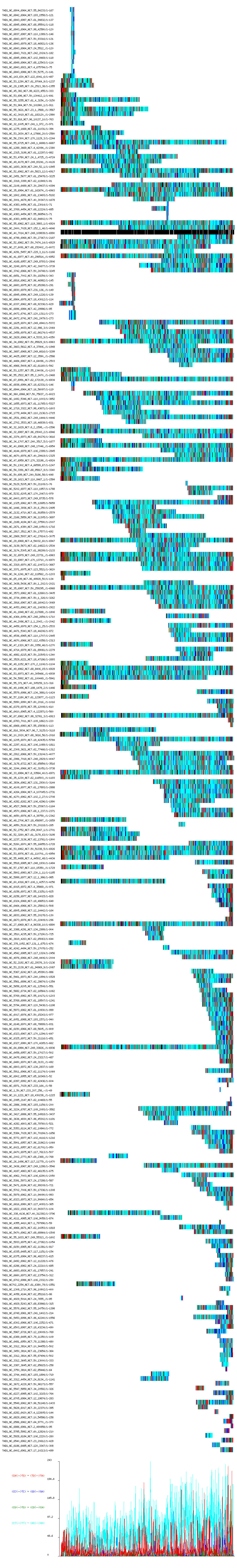

Supplement: Additional file 1 — Control Data. Compressed (.zip) file containing data relevant to control tests with Neurospora crassa Tad1 and 5S rDNA repeats, containing RIPCAL graphical (.png), tabular text (.txt) and fasta (.fas) alignments of repeat family matches. Also contains files (.png, .txt and .fas) for the MATE repeats from Aspergillus nidulans and Ty1 transposons from Microbotryum violaceum. [file 1471-2105-9-478-S1.zip › Supplementary Figure_Tad1_Neurospora_crassa.gif]

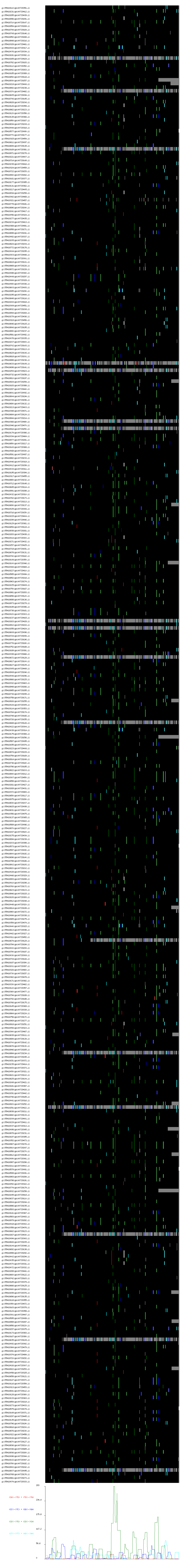

Supplement: Additional file 1 — Control Data. Compressed (.zip) file containing data relevant to control tests with Neurospora crassa Tad1 and 5S rDNA repeats, containing RIPCAL graphical (.png), tabular text (.txt) and fasta (.fas) alignments of repeat family matches. Also contains files (.png, .txt and .fas) for the MATE repeats from Aspergillus nidulans and Ty1 transposons from Microbotryum violaceum. [file 1471-2105-9-478-S1.zip › Supplementary Data_Ty1_copia_Microbotryum_violaceum_Popsetid_55418573.gif]
