# Supplementary material for: RIPCAL: a tool for alignment-based analysis of repeat-induced point mutations in fungal genomic sequences
Source: BMC Bioinformatics. 2008 Nov 12;9:478. doi: 10.1186/1471-2105-9-478 (PMC2621366; doi:10.1186/1471-2105-9-478)
Supplement: Additional file 3 — Supplementary Methods. Word (.doc) file explaining some of the methods in more detail. [file 1471-2105-9-478-S3.doc]

**Supplementary Methods:**

**Alignment based RIP analysis**

The RIPCAL tool does not perform alignment however can use a local installation of ClustalW. This is to provide an interface to an alignment tool most users are familiar with. Pre-aligned input may be the preferred choice of advanced users.

**Determining the model for comparison**

**By G:C content**

The sequence with the highest TOTAL G and C base counts is selected as the model (i.e. the sequence most likely to be the least RIP affected). This may not be the appropriate method of model selection if the aligned sequences are of variable lengths as a longer sequence is more likely to be chosen than shorter one.

**User defined**

If this option is selected, RIPCAL looks for attribute tags in the GFF input of form:

note=model (GFF3) OR,

note model (GFF2)

or if the pre-aligned input mode is selected, RIPCAL uses the first sequence listed in the Fasta or ClustalW format input as the model

Note: this may require you to cut and paste your desired model sequence to be first in the alignment file, converting your alignments to Fasta format first makes this a lot easier.

**By ‘Degenerate Consensus’**

This method determines the most common base at each position of the alignment (where sequence number > 2). Degenerate base letters are used where 2 or more base counts are equal. The degenerate consensus method assigns degenerate bases W, S, M K, R, Y, B, D, H, V or N:

| Degenerate base letter | Corresponds to |
| --- | --- |
| W | A/T |
| S | G/C |
| M | A/C |
| K | G/T |
| R | G/A |
| Y | T/C |
| B | G/T/C OR not A |
| D | A/G/T OR not C |
| H | A/C/T OR not G |
| V | G/C/A OR not T |
| N | A/C/G/T |

‘N’ is used in the degenerate consensus to refer to any base pair combination but is not assigned a probability of RIP mutation when calculating RIP mutation from a degenerate consensus.

Because each sequence in the alignment is now compared to an ambiguous consensus, mutation counts are converted to ‘probabilities of mutation’. The table below outlines the probability of nucleotide identity for each degenerate base letter:

| **1/1** | **1/2** | **1/3** |
| --- | --- | --- |
| A | M/R/W | D/H/V |
| C | M/S/Y | B/H/V |
| G | K/R/S | B/D/V |
| T | K/W/Y | B/D/H |

RIP probability is determined by the table above. i.e. for consensus dinucleotide MpD mutating to TpA in aligned sequences, there is a (1/2*1/3=1/6) chance that this is a CpA🡪TpA mutation.

In some cases this may be the most appropriate method to use, as it can detect RIP mutation among a repeat family of diverse sequence and RIP mutation profiles, as opposed to the highest G+C method, which chooses the sequence in the family most likely to be the least RIP affected. If the highest G+C sequence appears to be an anomaly compared to the majority of sequences, this method is a good choice.

**The RIPCAL visual output**

The alignment diagram is a visual representation of the alignment file received from ClustalW/pre-aligned input. Sequences appear in identical order to that of the alignment input. Usually this means that sequences are grouped according to similarity (default ClustalW alignment ordering).

If reference sequence selection methods are by G:C content or user-defined, one of these sequences will be represented in black and white only – i.e. indicating no sequence variation from itself (the reference). This may not be the case if the method chosen is degenerate consensus

The y-axis of the plot at the bottom of the output represents the overall frequency of RIP mutations (type indicated by colour) along a scanning window (default size 50bp) at each position of the alignment. This means that at alignment position ‘x’, the total RIP mutation counts are determined from all sequences in the alignment from position (x-24) to position (x+25). This can show the localised effects of RIP changes in discrete sequence regions.

**RIP index Scan**

This method is not used in the publication, but is included to make users aware of this feature. As RIP indices are the only method of detection of ancient/single copy RIP mutated sequences this method may be useful in some cases.

Additional (“non-published”) indices in this paper are suggested as alternatives, but not replacements to the previously published RIP indices TpA/ApT and (CpA+TpG)/(ApC+GpT). The rationale behind these new RIP indices is that the ratio of pre-RIP to post-RIP dinucleotides should be low in RIP mutated sequences. By comparison of these four indices it is also possible to detect non-conventional (ie not CpA🡪TpA) dinucleotide bias (which the previous indices are designed to target).

RIPCAL breaks down long sequences into smaller chunks (default chunk size 200bp). If these small sub-regions are above the threshold for RIP for the selected RIP indices, these chunks are stored in memory. Overlapping RIP-affected chunks are merged into longer regions, which are subject to a minimum size threshold of 300bp (default).

**RIP analysis of *S. nodorum de novo* repeat families**

The accessions and versions of scaffolds of the *S. nodorum* genome assembly used in this publication are as follows:

| Scaffold# | NCBI Accession | NCBI version | Length |
| --- | --- | --- | --- |
| 1 | CH445325 | 1 | 2531949 |
| 2 | CH445326 | 1 | 1644489 |
| 3 | CH445327 | 1 | 1614665 |
| 4 | CH445328 | 1 | 1598912 |
| 5 | CH445329 | 1 | 1595170 |
| 6 | CH445330 | 2 | 1480485 |
| 7 | CH445331 | 1 | 1445540 |
| 8 | CH445332 | 1 | 1399599 |
| 9 | CH445333 | 1 | 1333432 |
| 10 | CH445334 | 1 | 1319089 |
| 11 | CH445335 | 1 | 1275933 |
| 12 | CH445336 | 1 | 1271674 |
| 13 | CH445337 | 1 | 1045363 |
| 14 | CH445338 | 1 | 1021686 |
| 15 | CH445339 | 1 | 1005965 |
| 16 | CH445340 | 1 | 976000 |
| 17 | CH445341 | 1 | 970256 |
| 18 | CH445342 | 1 | 891369 |
| 19 | CH445343 | 1 | 830519 |
| 20 | CH445344 | 1 | 748259 |
| 21 | CH445345 | 1 | 716313 |
| 22 | CH445346 | 1 | 578534 |
| 23 | CH445347 | 1 | 571407 |
| 24 | CH445348 | 1 | 552433 |
| 25 | CH445349 | 2 | 494812 |
| 26 | CH445350 | 1 | 483355 |
| 27 | CH445351 | 1 | 476925 |
| 28 | CH445352 | 1 | 471284 |
| 29 | CH445353 | 1 | 441516 |
| 30 | CH445354 | 1 | 429568 |
| 31 | CH445355 | 1 | 415093 |
| 32 | CH445356 | 1 | 391842 |
| 33 | CH445357 | 1 | 383353 |
| 34 | CH445358 | 1 | 371589 |
| 35 | CH445359 | 1 | 365181 |
| 36 | CH445360 | 1 | 328239 |
| 37 | CH445361 | 1 | 320110 |
| 38 | CH445362 | 1 | 312146 |
| 39 | CH445363 | 1 | 308193 |
| 40 | CH445364 | 1 | 306474 |
| 41 | CH445365 | 1 | 299810 |
| 42 | CH445366 | 1 | 245939 |
| 43 | CH445367 | 1 | 226755 |
| 44 | CH445368 | 1 | 223268 |
| 45 | CH445369 | 1 | 212241 |
| 46 | CH445370 | 1 | 142652 |
| 47 | CH445371 | 1 | 142484 |
| 48 | CH445372 | 1 | 140753 |
| 49 | CH445373 | 1 | 128119 |
| 50 | CH445374 | 1 | 88068 |
| 51 | CH445375 | 1 | 74634 |
| 52 | CH959327 | 1 | 57507 |
| 53 | CH445376 | 1 | 38892 |
| 54 | CH959328 | 1 | 37858 |
| 55 | CH445377 | 1 | 32376 |
| 56 | CH959329 | 1 | 32169 |
| 57 | CH445378 | 1 | 29276 |
| 58 | CH445379 | 1 | 26957 |
| 59 | CH959330 | 1 | 23504 |
| 60 | CH445380 | 1 | 20941 |
| 61 | CH445381 | 1 | 19523 |
| 62 | CH445382 | 1 | 16634 |
| 63 | CH445383 | 1 | 16108 |
| 64 | CH445384 | 1 | 14656 |
| 65 | CH445385 | 1 | 14003 |
| 66 | CH445386 | 1 | 19742 |
| 67 | CH445387 | 1 | 12017 |
| 68 | CH445388 | 1 | 10721 |
| 69 | CH959331 | 1 | 9585 |
| 70 | CH445389 | 1 | 10534 |
| 71 | CH445390 | 1 | 10198 |
| 72 | CH959332 | 1 | 7904 |
| 73 | CH445391 | 1 | 8577 |
| 74 | CH959333 | 1 | 7355 |
| 75 | CH445392 | 1 | 6994 |
| 76 | CH445393 | 1 | 8327 |
| 77 | CH959334 | 1 | 5582 |
| 78 | CH445394 | 1 | 6050 |
| 79 | CH959335 | 1 | 4869 |
| 80 | CH959336 | 1 | 4815 |
| 81 | CH959337 | 1 | 4795 |
| 82 | CH959338 | 1 | 4630 |
| 83 | CH959339 | 1 | 3428 |
| 84 | CH959340 | 1 | 3262 |
| 85 | CH959341 | 1 | 3153 |
| 86 | CH959342 | 1 | 3078 |
| 87 | CH959343 | 1 | 2902 |
| 88 | CH959344 | 1 | 2893 |
| 89 | CH959345 | 1 | 2874 |
| 90 | CH959346 | 1 | 2796 |
| 91 | CH959347 | 1 | 2776 |
| 92 | CH959348 | 1 | 2693 |
| 93 | CH959349 | 1 | 2574 |
| 94 | CH959350 | 1 | 2560 |
| 95 | CH959351 | 1 | 2514 |
| 96 | CH959352 | 1 | 2504 |
| 97 | CH959353 | 1 | 2488 |
| 98 | CH959354 | 1 | 2445 |
| 99 | CH959355 | 1 | 2445 |
| 100 | CH959356 | 1 | 2360 |
| 101 | CH959357 | 1 | 2329 |
| 102 | CH959358 | 1 | 2322 |
| 103 | CH959359 | 1 | 2317 |
| 104 | CH959360 | 1 | 2160 |
| 105 | CH959361 | 1 | 2149 |
| 106 | CH959362 | 1 | 2141 |
| 107 | CH959363 | 1 | 2015 |
| 108 | CH959364 | 1 | 2011 |
| 109 | CH959365 | 1 | 2005 |

**Genomic match coverage**

“Genomic match coverage”, in the methods section refers to the sequence copy number over an alignment of genomic matches to the repeat consensus. At alignment termini, where this coverage falls below 10 these alignments have been truncated to the point where coverage equals or exceeds 10. This condition was applied after RepeatScout results were obtained to ensure that the repeat set was representative of highly repetitive sequences.

**RIP dominance**

**CpA↔TpA dominance is calculated by**

$\overline{\left(\frac{(CpA\leftrightarrow TpA)}{(CpC\leftrightarrow TpC)+(CpG\leftrightarrow TpG)+(CpT\leftrightarrow TpT)}\right)}$ (8)

**CpC↔TpC dominance is calculated by**

$\overline{\left(\frac{(CpC\leftrightarrow TpC)}{(CpA\leftrightarrow TpA)+(CpG\leftrightarrow TpG)+(CpT\leftrightarrow TpT)}\right)}$ (9)

**CpG↔TpG dominance is calculated by**

$\overline{\left(\frac{(CpG\leftrightarrow TpG)}{(CpA\leftrightarrow TpA)+(CpC\leftrightarrow TpC)+(CpT\leftrightarrow TpT)}\right)}$ (10)

**CpT↔TpT dominance is calculated by**

$\overline{\left(\frac{(CpT\leftrightarrow TpT)}{(CpA\leftrightarrow TpA)+(CpC\leftrightarrow TpC)+(CpG\leftrightarrow TpG)}\right)}$ (11)

**Why is there a bidirectional arrow between mutated dinucleotides in the RIP dominance statistic (e.g. CpA↔TpA)?**

When comparing repeat families as was the case with S. nodorum in this publication, it is not known which sequences are truly less/more RIP affected. In fact, all repeats within a genome are likely to interact with each other and become affected by RIP. Therefore CpN mutations in both directions are counted. Counting RIP mutations in a single direction would be appropriate if the repeat alignment contained a known precursor repeat (e.g. from a different organism) as the comparison model. Note: While bidirectional counts are used in the graphical outputs, directional RIP mutation counts of alignments are provided in the RIPCAL tabular output.
